# The relationship between benevolent childhood experiences and depression among Chinese university students: the serial mediating role of family relationships and sleep quality

**DOI:** 10.3389/fpubh.2025.1450932

**Published:** 2025-02-25

**Authors:** Chong Li, Yihan Wang, Jingjing Wang, Yuhao Wang, Yunjiao Luo, Na Yan, Yingxue Wang, Guixiang Sun, Ying Zhang, Wei Wang

**Affiliations:** ^1^Graduate School, Xuzhou Medical University, Xuzhou, China; ^2^School of Public Health, Xuzhou Medical University, Xuzhou, Jiangsu, China; ^3^Huaian Center for Disease Control and Prevention, Huai’an, China

**Keywords:** benevolent childhood experiences, depression, family relationships, sleep quality, serial mediation

## Abstract

**Introduction:**

Depression represents a significant mental health challenge among university students. Previous studies have revealed a relationship between benevolent childhood experiences (BCEs) and depression, but the roles of family relationships and sleep quality in mediating the link between BCEs and depression remain unclear. This study constructed a serial mediating model to examine whether family relationships and sleep quality mediated the relationship between BCEs and depression among Chinese university students.

**Methods:**

A total of 1830 university students from 25 universities in three provinces of China got recruited in this study. The assessment utilized the Benevolent Childhood Experiences Scale (BCEs-10) for childhood experiences, the Quality of Family Relationships Scale for family dynamics, the Pittsburgh Sleep Quality Index (PSQI) single-item for sleep quality, and the Center for Epidemiological Survey Depression Scale (CES-D-10) for depression. Correlation analyses and serial mediation modeling were conducted using SPSS 25.0 with PROCESS macro v3.4.1.

**Results:**

BCEs, family relationships, and sleep quality scores were all found to be negatively correlated with depression scores (*r* = −0.46, −0.32, −0.47, respectively, all *p* < 0.01). Family relationships, and sleep quality scores were positively correlated with BCEs scores (*r* = 0.31, 0.27, respectively, both *p* < 0.01). There was a positive correlation between the family relationships score and sleep quality score (*r* = 0.22, *p* < 0.01). Mediating analysis indicated that BCEs had a direct effect on depression (the direct effect accounted for 71.54%). Depression was affected by BCEs partly through three different pathways: the mediating role of family relationships (the mediation effect accounted for 8.50%), the mediating role of sleep quality (the mediation effect accounted for 16.40%), and the serial mediating role of both family relationships and sleep quality (the serial mediation effect accounted for 3.56%).

**Discussion:**

The findings of this study demonstrated that family relationships and sleep quality partially mediated the association between BCEs and depression by serial mediating effects.Thus, improving sleep quality and family intervention may be effective measures to protect Chinese university students from depression.

## Introduction

Depression is one of the most common psychological problems experienced by university students (1), a systematic review reported that depression rates ranged from 10 to 85% with an average prevalence of 30.6% (2). Recent studies have highlighted that university students are particularly vulnerable to mental health challenges due to multiple stressors including academic pressure, adjustment to new environments, financial concerns, and future career uncertainty (3). Longitudinal studies show that this vulnerability has been further exacerbated in recent years, with studies showing increasing trends in depression rates among university students globally (4). Depressive symptoms lead to adverse consequences, such as poorer academic achievements, relationship instability, and suicidal ideation in university students (5–7). Additionally, research has shown that untreated depression during university years can have long-lasting effects on students’ personal health, such as sleep quality, strained family relationships, and future career development (8). Therefore, reducing the risk of depression among university students is crucial, and understanding the underlying factors contributing to depression is essential.

A diversity of factors has been reported to be associated with depression in university students, one of which was family relationships (8, 9). University students represent a particularly vulnerable population with regard to mental health challenges, as they navigate the complex transition to adulthood while managing academic pressures, social adjustments, and identity formation (3). The quality of family relationships acted a crucial role in adolescents’ psychological adjustment. This vulnerability is often exacerbated during the university years, as students experience unprecedented autonomy while simultaneously maintaining complex familial bonds and dependencies (8, 10). As the core of the family, the quality of the parental relationship cannot be ignored in the psychology and behavior of university students. Researchers had found that children in a divorced family may exhibit depressive symptoms (11). Similarly, those who lived in a hostile family environment or family conflicts were more likely to experience depressive symptoms (9). Whereas, a study conducted on 2,436 university students reported that living with parents and family closeness were protective factors for depression (12).

Besides, good sleep quality is an important sign of physical and mental health (13). The relationship between sleep quality and depression has been well-documented in the general population (14, 15). Evidence suggests that reduced sleep quantity and quality might be a factor in mental health issues (16). A study found that up to 90% of people with diagnosed depression had trouble sleeping (17). Furthermore, students who had poor sleep quality were more likely to increase depression risk (18). Research indicates that the prevalence of poor sleep quality among university students is twice as high as that of the general population, with 73.5% of university students experiencing sleep disturbances (19). This increased prevalence, combined with the developmental sensitivity of young adulthood, makes the university student population especially vulnerable to sleep-related mental health complications (20). In addition, good sleep quality was closely related to family relationships (21–23).

Benevolent childhood experiences (BCEs), also known as positive childhood experiences (PCEs) or advantageous childhood experiences (counter-ACEs) in some studies (24, 25), refer to the beneficial experiences that occurred under the age of 18, including parent–child attachment, positive parenting (e.g., responsiveness, support, and parental warmth), family health, and positive relationships with friends, in school, and the community (26). Studies found that BCEs were related to mental health among university students (27, 28). For instance, one study in the US showed that respondents who grew up in a happy family had significantly higher levels of cognitive functioning (29). BCEs also contributed to the prevention of depression later in life and laid the groundwork for better family health in adulthood (30, 31). At present, current studies mainly focused on the influence of adverse childhood experiences (ACEs) on depression (32–34), and few have found the importance of the relationship between BCEs and depression.

Previous work had separately reported the associations of BCEs, family relationships, and sleep quality with depression (9, 35, 36). Despite these individual associations, there are significant gaps in our understanding of how these factors interact to influence depression among university students. To address this, the present study draws upon the Mindsponge Theory, a recent psychological theory of information processing, to explore these complex relationships (37). The Mindsponge Theory conceptualizes the mind as an information processing system that filters and integrates information like a sponge based on core values and beliefs, where early life experiences like BCEs shape fundamental cognitive schemas and filtering mechanisms that determine how individuals process and interpret environmental information, ultimately influencing their vulnerability to depression (37, 38). From a cognitive perspective, these early-established processing patterns influence attention, interpretation, and memory biases that are characteristic of depression (39). BCEs tend to establish stable, low-entropy processing patterns that promote adaptive cognitive strategies and emotional regulation. In contrast, negative experiences may create unstable, high-entropy patterns that lead to negative cognitive biases and maladaptive emotion regulation strategies (40). Family relationships serve as crucial ongoing information exchange systems that can either reinforce or modify these early established patterns (37). Current family interactions provide new information that is processed through existing cognitive filters while potentially updating those same filters (41). Additionally, sleep quality acts as a critical mechanism for information reorganization and entropy reduction (42). Adequate sleep allows for proper cognitive processing and emotion regulation, while poor sleep may exacerbate negative cognitive biases and information processing difficulties (43).

**Figure 1:**
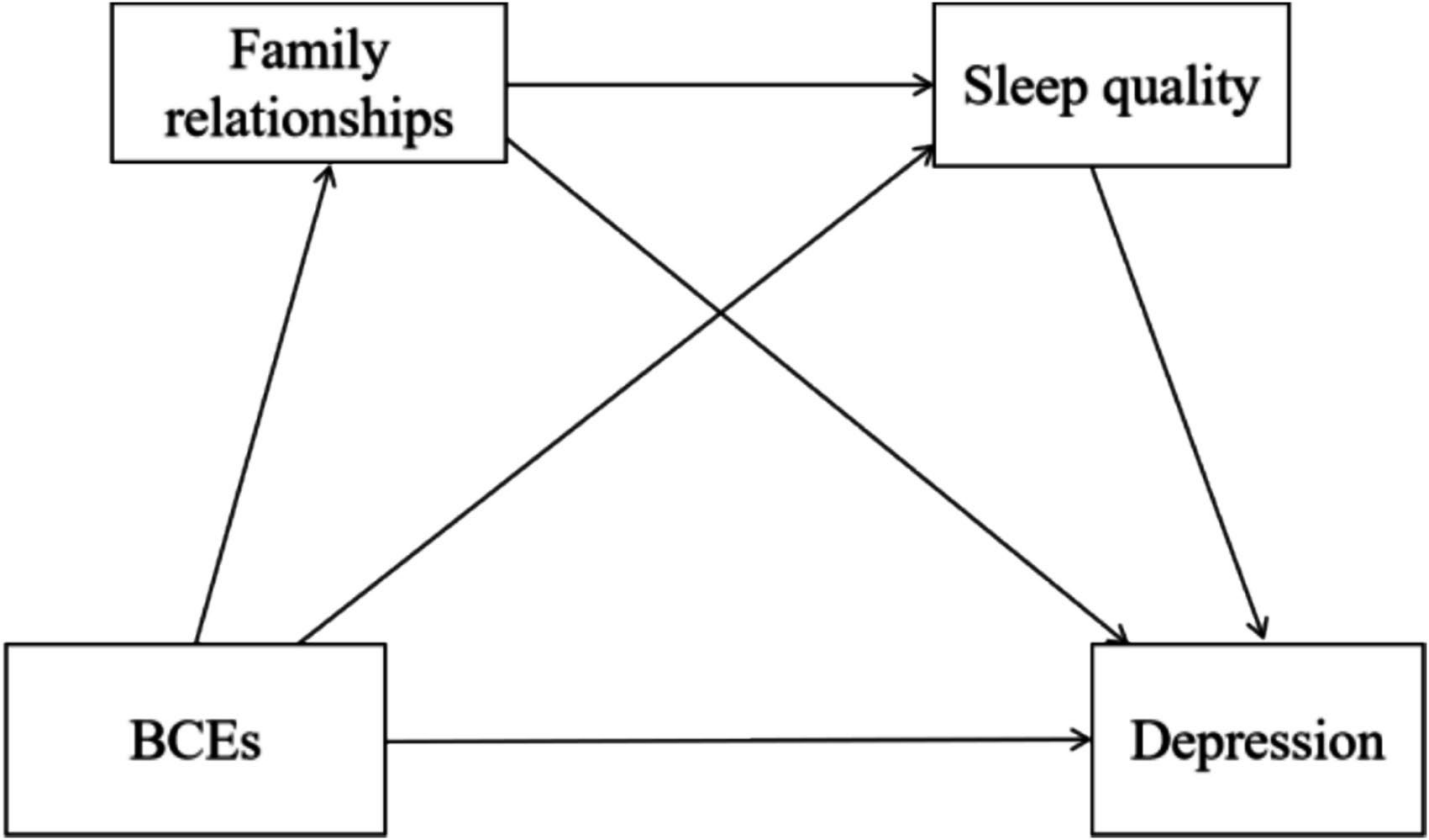
Hypothesized relationships among BCEs, family relationships, sleep quality, and depression.

Given the high prevalence of depression among university students and the need for evidence-based interventions, this study aims to examine the complex interplay between BCEs and depression, building on this theoretical framework to investigate potential mediating mechanisms. Specifically, we investigate whether family relationships and sleep quality serve as mediating pathways, both independently and sequentially, in the relationship between BCEs and depression. As conveyed in Figure 1, we hypothesized that (1) BCEs can directly and negatively predict depression in university students; (2) Both family relationships and sleep quality can play a mediating role between BCEs and depression; (3) Family relationship and sleep quality play a serial mediating role between BCEs and depression.

## Methods

### Participants and procedure

A cross-sectional study was conducted using a combination of random sampling and cluster sampling methods. Three cities (Xuzhou, Nanjing, and Wuhan) were selected in China. Based on the size of each city, we determined the number of universities to be sampled and randomly selected two universities per city. Within each university, three colleges were randomly selected. The cluster sampling method was then applied to select two to three classes from each grade level within the selected colleges. Data collection was carried out by trained research assistants, who distributed the online questionnaire via WeChat using the Questionnaire Star platform. Before initiating the survey, the assistants explained the study’s purpose, ensured participants’ understanding of their rights, and confirmed their informed consent. Measures were taken to ensure the quality of the responses, including: (1) excluding questionnaires with logical errors (e.g., contradictory answers); (2) excluding responses completed in less than 600 s, as this was deemed insufficient time to provide thoughtful answers; and (3) conducting pilot testing to refine the survey instrument for clarity and usability.

The survey took approximately 30 min to complete for most participants. The principles of anonymity and voluntariness were upheld throughout the process. Data were securely stored in a password-protected database accessible only to the research team. The raw data underwent thorough cleaning and validation to ensure completeness and consistency before analysis.

A total of 2022 students were interviewed through the online questionnaire from March 2021 to May 2021. A sample of 1830 was used to analyze after excluding the individuals with incomplete data for key variables (response rate: 90.50%). The demographic variables were shown in Table 1. The mean age of the participants was 20.01 years old (range 17–24). Approximately 69.45% of the participants were females, and 45.41% (*n* = 298) were sophomores. In addition, over half of the sample was not the only child in their families, and most of the participants had a monthly living expense of 1,000–2000 RMB (79.13%).

**Table 1:** Sociodemographic characteristics of the sample (*N* = 1830).

Variables	Category	N	%
Gender	Male	559	30.55
	Female	1,271	69.45
Age	<20	556	30.38
	≥20	1,274	69.62
Grades	First-year undergraduate	279	15.25
	Second-year undergraduate	831	45.41
	Third-year undergraduate	668	36.50
	Fourth-year undergraduate	52	2.84
Only child	Yes	914	49.95
	No	916	50.05
Residence status	Urban	1,035	56.56
	Rural	795	43.44
Monthly living expenses (RMB)	≤1,000	93	5.08
	1,001–2000	1,448	79.13
	2001–3,000	227	12.40
	>3,000	62	3.39

### Ethical considerations

Approval was obtained from the ethics committee of the Xuzhou Center for Disease Control and Prevention and Xuzhou Medical University. The procedures used in this study adhere to the tenets of the Declaration of Helsinki. All participants gave their written informed consent before their inclusion in the study.

### Measures

#### Common method biases tests and preliminary analyses

To address potential common method bias from single-source data collection, we implemented several preventive measures. First, we ensured participant anonymity and emphasized the importance of truthful responses during data collection. Second, we planned to use Harman’s single-factor test (44) to assess common method variance. The Harman’s single-factor test results revealed five factors with initial eigenvalues greater than 1, with the first factor accounting for 27.70% of the total variance. This percentage was well below the 40% threshold, indicating that common method bias was not a significant concern in this study and supporting the credibility of our data. Prior to conducting the main analyses, we performed comprehensive data screening procedures. No missing values and outliers were identified in our dataset. The normality of data distribution was assessed using the Kolmogorov–Smirnov test, while homoscedasticity was evaluated through Levene’s test. These preliminary analyses confirmed that our data met the necessary statistical assumptions for the subsequent analyses.

**Demographic characteristics** such as age (in years), residence status (urban/rural), gender (male/female), grade in years (First-year undergraduate, Second-year undergraduate, Third-year undergraduate, Fourth-year undergraduate), only child (yes/no), and monthly living expenses (1,000/1001–2000/2001–3,000/>3000RMB) were obtained via self-report.

**BCEs** were evaluated using the Benevolent Childhood Experiences Scale, which has been validated in Chinese cultural contexts. It is a 10-item checklist of benevolent experiences between ages 0–18 years (25). An example item was “Did you have a chance to enjoy yourself?” Each item consists of two responses: “yes” and “no.” The “Yes” response was scored as 2 and the “no” was scored as 1. All items were summed to obtain an overall BCEs score. The higher the score, the greater the BCEs. Cronbach’s *α* was 0.73 in this study.

**Depression** was measured using the Center for Epidemiological Survey, Depression Scale (CES-D). The CESD-10, validated in Chinese populations, is a short version of the CESD-20 (45, 46), was administered to assess depressive symptoms experienced during the past week. This 10-item questionnaire utilized a 4-point Likert scale: 1, rarely or no time (<1d); 2, some or a little of the time (1-2d); 3, from time to time (3-4d); 4, all the time (5–7d). Items 5 and 8 were scored reversely. All graded items were summed up to provide a total score, with higher scores indicating greater frequency of depression experienced over the past week. Cronbach’s *α* was 0.87 in this study.

**Family relationships** were tested with the Quality of Family Relationships Scale, which was developed by Zhao (47). This validated scale measured the mother–child relationship, father-child relationship, and parental relationship. The scale comprised four items and employed a 4-point scale, ranging from “terrible” (1 point) to “good” (4 points). Total scores were calculated by summing up four items scores for analysis. Higher scores indicated better family relationships. Cronbach’s *α* was 0.87 in this study.

**Sleep quality** was assessed using a culturally validated single item from the Pittsburgh Sleep Quality Index(PSQI) (48). Participants were asked to answer the following question: “How did you judge your sleep quality in the past month?” The item employed a 4-point scale, ranging from “worst” (1 point) to “best” (4 points). Higher scores indicate better sleep quality.

### Statistical analyses

The macro software process version 3.4.1, based on SPSS 25.0, was used to analyze the mediation effect. An analysis of demographic characteristics was performed using descriptive statistics. First, Spearman’s correlation analysis was employed to examine the relationships between BCEs, family relationships, sleep quality, and depression. Second, the mediated models were used to examine the potential mechanisms between BCEs, family relationships, sleep quality, and depression by Haye’s PROCESS macro for SPSS 25.0. The serial mediation model was first employed to investigate the sequential mediating pathway from BCEs through family relationships to sleep quality and ultimately to depression. For the serial mediation analysis, we established three equations to test the mediating role of family relationships and sleep quality in the relation between BCEs and depression, with gender, age, an only child, and family location as control variables. To further verify the relative importance of these two mediators, we also conducted a parallel mediation analysis. Different from the serial mediation model that assumes a sequential path, the parallel model examines the concurrent and independent mediating effects of family relationships and sleep quality between BCEs and depression, which allows us to compare the magnitude of their individual mediating effects. Through these analyses, we tested the mediating roles of family relationships and sleep quality in the connection between BCEs and depression, and determined their relative importance in this relationship. In the mediated model, age, residence status, gender, and only child were considered covariates. All variables were centralized before the analysis. A probability of *p* < 0.05 was considered statistically significant.

## Results

### Bivariate correlations

As shown in Table 2, BCEs, family relationships, and sleep quality scores were negatively correlated with depression (*r* = −0.51, −0.33, −0.49, respectively, all *p* < 0.01). Family relationships and sleep quality scores were positively correlated with BCEs scores (*r* = 0.33, 0.28, respectively, both *p* < 0.01). There was a positive correlation between the family relationships score and sleep quality score (*r* = 0.22, *p* < 0.01).

**Table 2:** Descriptive statistics and correlations between study variables (*N* = 1830).

	1	2	3	4
1.BCEs	1			
2.family relationships	0.33**	1		
3.sleep quality	0.28**	0.22**	1	
4.depression	−0.51**	−0.33**	−0.49**	1
Mean	18.67	13.93	3.05	17.58
SD	1.78	2.21	0.75	5.92
Minimum value	10.00	5.00	1.00	10.00
Maximum value	20.00	16.00	4.00	40.00

*p < 0.05, **p < 0.01.

### The serial mediating effects analysis

Table 3 indicated that BCEs (*β* = 0.33, *p* < 0.001) had a direct prediction on the level of family relationships in equation1. Moreover, equation 2 showed BCEs (*β* = 0.23, *p* < 0.001) and family relationships (*β* = 0.15, *p* < 0.001) could directly predict the level of sleep quality. Apart from the above, equation 3 demonstrated BCEs (*β* = −0.36, *p* < 0.001), family relationships (*β* = −0.13, *p* < 0.001) and sleep quality (*β* = −0.36, *p* < 0.001) had a negative prediction effect on depression.

**Table 3:** Testing the mediating role of family relationships and sleep quality in the relationship between BCEs and depression in multiple linear regression results.

Predictor variable	Outcome variable	R^2^	F	*β*	*t*	Boot LLCI	Boot ULCI
Equation 1							
BCEs	Family relationships	0.11	44.92	0.33	14.83	0.29	0.37
Gender				0.03	0.55	−0.07	0.12
Age				0.02	0.35	−0.08	0.11
Only child				−0.05	−1.10	−0.14	0.04
Family location				−0.01	−0.15	−0.10	0.08
Equation 2							
BCEs	Sleep quality	0.10	33.77	0.23	9.65	0.18	0.27
Family relationships				0.15	6.32	0.10	0.19
Gender				0.06	1.13	−0.04	0.15
Age				−0.11	−2.27	−0.21	−0.02
Only child				−0.03	−0.74	−0.13	0.06
Family location				−0.05	−1.18	−0.15	0.04
Equation 3							
BCEs	Depression	0.40	177.12	−0.36	−18.45	−0.40	−0.32
Family relationships				−0.13	−6.75	−0.17	−0.09
Sleep quality				−0.36	−19.08	−0.40	−0.33
Gender				−0.02	−0.57	−0.10	0.06
Age				0.00	0.09	−0.07	0.08
Only child				0.05	−1.32	−0.12	0.03
Family location				−0.05	−1.29	−0.12	0.03

All the variables used in this table were centralized. Boot LLCI and Boot ULCL were a 95% confidence interval lower and a 95% confidence interval upper calculated by the bias-corrected percentile bootstrap method for testing indirect effects. 95% confidence intervals do not overlap with zero.

**Figure 2:**
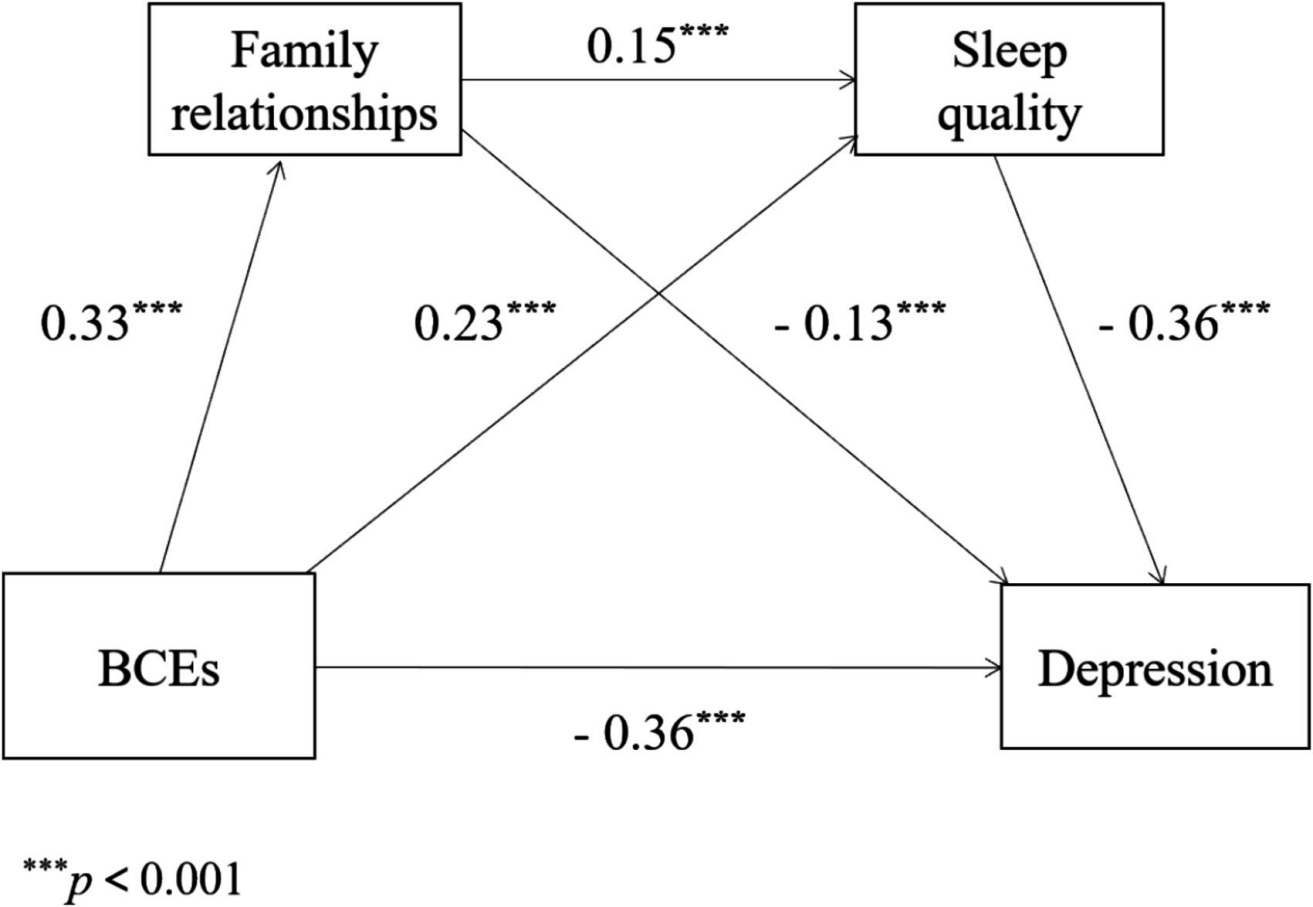
The serial mediating effect of BCEs and depression.

Table 4 and Figure 2 showed the results of the serial mediating effect of family relationships and sleep quality. It turned out that the total indirect effect was −0.144, which performed 28.46% of the total effect (−0.506) in the relationship between BCEs and depression. Particularly, three different pathways constituted the total indirect effect: BCEs influenced depression separately through family relationships and sleep quality, and through the serial mediating role of both family relationships and sleep quality, which were shown in the indirect effects 1, 2, and 3, respectively. In addition, indirect effects 1, 2, and 3 make up 8.50, 16.40, and 3.56% of the total effect respectively, and the 95% confidence intervals did not overlap with zero, which indicated that all indirect effects were negative significantly.

**Table 4:** The indirect effect of family relationships and sleep quality (*N* = 1830).

	Effect	Boot SE	Boot LLCI	Boot ULCI	Ratio of indirect to total effect
Total indirect effect	−0.14	0.01	−0.17	−0.12	28.46%
Indirect effect 1 (BCEs→ family relationships → depression)	−0.04	0.01	−0.06	−0.03	8.50%
Indirect effect 2 (BCEs → sleep quality → depression)	−0.08	0.01	−0.10	−0.06	16.40%
Indirect effect 3 (BCEs → family relationships → sleep quality→ depression)	−0.02	0.00	−0.03	−0.01	3.56%

Boot SE, Boot LLCI, and Boot ULCL were estimated standard error, 95% confidence interval lower and 95% confidence interval upper through bias–corrected percentile bootstrap method used for testing indirect effects. 95% confidence intervals did not overlap with zero.

### Parallel mediating effects of family relationships and sleep quality between BCEs and depression

**Figure 3:**
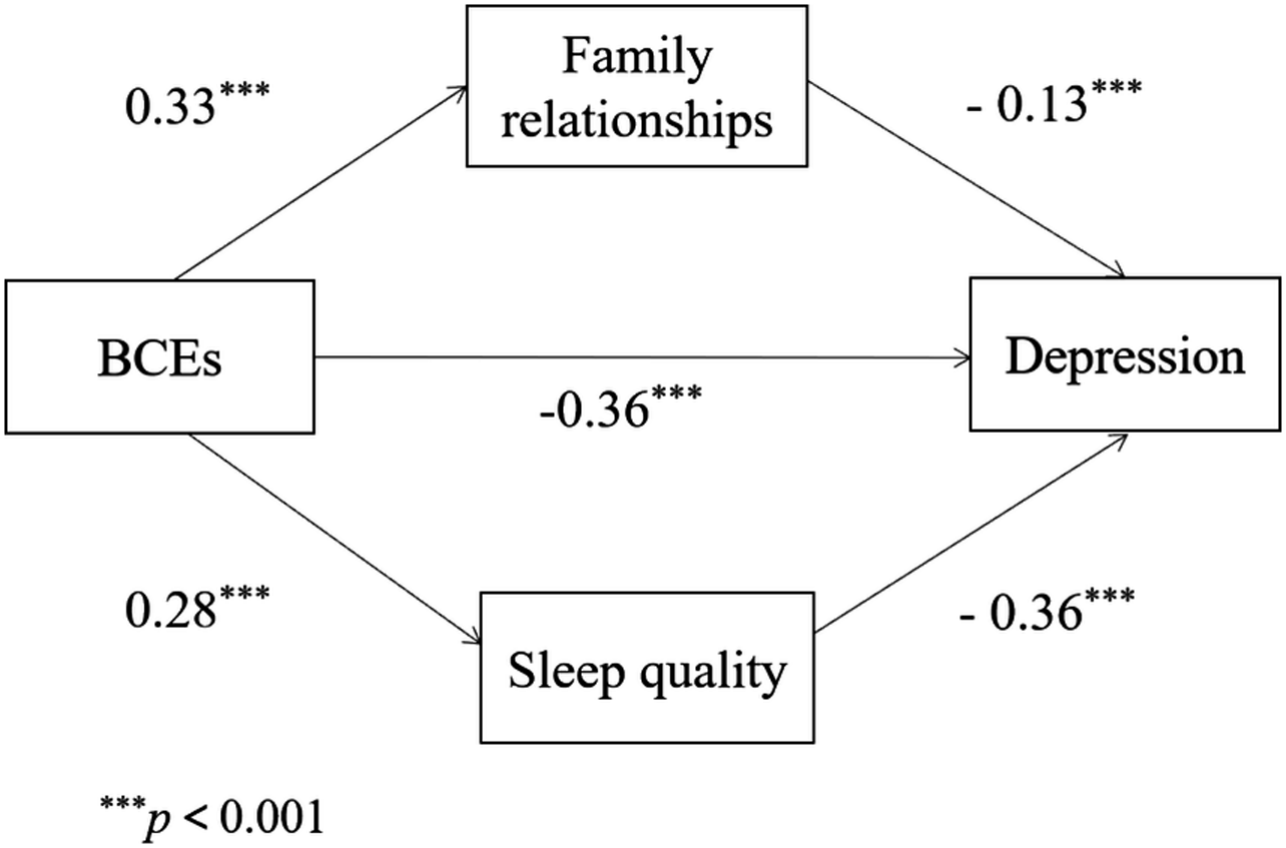
The mediating effect of family relationships and sleep quality in a parallel model.

The results were shown in Figure 3, we found the indirect effect of family relationships was −0.043, which accounted for 8.50% of the total effect (−0.506) in the relationship between BCE and depression. The 95% confidence interval was −0.060–−0.028. The indirect effect of sleep quality was −0.101, which performed 19.96% of the total effect (−0.506) in the relationship between BCEs and depression. The 95% confidence interval was −0.122–−0.081, and it did not overlap with zero, which meant that all indirect effects were negative significantly. Sleep quality acted a more important role than family relationships in that the mediating effect of sleep quality (−0.101) was higher than family relationships (−0.043).

## Discussion

This study explored the demographic features and the relationship between BCEs and depression among Chinese university students. Consistent with our hypothesis, it was found that BCEs affected depression by three pathways: family relationships, sleep quality, and the serial mediating effect of family relationships, and sleep quality.

Direct effects analysis indicated that BCEs predict a lower risk of depression among Chinese university students, aligning with previous research (25, 49) that has observed similar associations between BCEs and reduced depression, perceived stress, and loneliness. The reason may be that positive memory declined depression by reducing negative self-awareness of negative life events (50). The Mindsponge theoretical framework, established by Vuong and Napier (51), conceptualizes cognitive information processing as a dynamic filtering mechanism that evaluates incoming information against established core values. This framework posits that individuals process information through systematic cost–benefit analyses based on compatibility with their existing value system (52). BCEs serve as fundamental reference points for evaluating and responding to life events, creating a robust “filtering mechanism” that helps individuals process negative information more adaptively (51). BCEs included positive examples, emotional support, and childhood family stability (31). Students with abundant BCEs demonstrate enhanced information processing capabilities, wherein their established cognitive framework effectively integrates positive stimuli while modulating negative experiences through reference to internalized positive values and beliefs. This mechanism facilitates greater confidence and resilience in managing interpersonal relationships (53, 54) and mental health challenges (55–58). This mechanism is particularly significant in Chinese society, where strong family bonds and collective well-being are deeply valued cultural traditions that can significantly influence mental health outcomes (59).

We found that there was a serial mechanism between BCEs and depression in university students. First, the results showed that family relationships partially mediated the relationship between BCEs and depression. Children that receive more emotional support from their families and social networks may have better long-term mental health outcomes and fewer chronic health problems as they grow up, which becomes their valuable family health resources (60). Additionally, positive parenting factors and behaviors (such as connectedness, bonding, involvement, and support) may serve to promote mental health and decline depression risk ultimately (61). This is especially significant in Chinese culture, where family interdependence and mutual support are fundamental values that shape individuals’ psychological development (62). According to Mindsponge theory, positive family relationships developed through BCEs create a supportive “trust filter” that helps individuals better evaluate and cope with stressful situations (52). When university students are faced with negative life events, they seek help from family members or within their social network for additional emotional support, which can alleviate high levels of stress and reduce the risk of depression. Second, poor sleep quality emerged as a significant predictor of depression (63). Those with more BCEs develop better “filtering mechanisms” for managing stress and maintaining regular sleep patterns, thereby reducing their risk of depression (51). Sleep quality served as a more direct “processing unit” within the Mindsponge framework, as evidenced by its stronger mediating effect (−0.101) compared to family relationships (−0.043) in parallel mediation model, demonstrating how BCEs influence individuals’ sleep patterns and, consequently, their vulnerability to depression (64).

While both serial mediation model and parallel mediation model provide valuable insights, we emphasize the serial mediating model as it better captures the sequential mechanism of information processing in the Mindsponge framework. This sequential relationship demonstrates how BCEs first enhance the “trust filter” of family relationships, which then strengthens the “processing unit” of sleep quality regulation, ultimately reducing depression risk through improved emotional security and stress management capabilities (65).

These findings have important clinical implications for mental health professionals working with university students. First, our results suggest that strengthening family relationships could be a crucial intervention target for preventing and treating depression among university students. Mental health services could develop family-based interventions that enhance communication and emotional support within families. In the Chinese educational context, where family involvement is both expected and valued, such interventions could be particularly effective when integrated into existing university support systems. Second, sleep hygiene education and intervention programs should be integrated into university mental health services, as improving sleep quality appears to be a vital pathway for reducing depression risk. Finally, mental health professionals should consider assessing and leveraging students’ positive childhood experiences as potential protective factors when developing treatment plans. This approach aligns well with Chinese cultural values that prioritize family harmony and educational success, creating a comprehensive framework for supporting student mental health.

### Strengths and limitations

This study discussed for the first time the intrinsic mechanism of BCEs and depression in Chinese university students. However, several limitations must be noted in this study. (1) The study population was heterogeneous, as these university students from different colleges may experience varying levels of academic stress and challenges, potentially affecting the generalizability of results. (2) The typical university age range coincides with a recognized period of increased vulnerability to mental illness onset due to both genetic and environmental factors, which could influence our findings. (3) Given the cross-sectional nature of our study, we cannot determine causal relationships between variables. Future research should utilize experimental or longitudinal designs to assess the proposed mediating models. (4) All variables were based on subjective reporting, which may introduce reporting bias. (5) Sleep quality was measured with a single item, which may limit the reliability of the results. Future studies could adopt more comprehensive and validated scales to verify these findings.

## Conclusion

To sum up, this study found that family relationships and sleep quality played a serial mediation role in the relationship between BCEs and depression, and some measures can be taken to prevent the occurrence of depression among Chinese university students. On one hand, we encourage parents to timely understand the psychological state of university students and provide the corresponding emotional support. Healthy family relationships may be especially beneficial for students in developing positive familial, interpersonal, and other social relationships, as well as developing appropriate emotion regulation capacities and promoting mental health.

On the other hand, we found that the mediating effect of sleep quality suggested that university institutions should recognize the importance of sleep hygiene and educate students to develop good habits, some of them may not be aware of the importance of this issue and subconsciously believe that sleeping late is just a nocturnal culture. Furthermore, parallel models show that sleep quality played a more important role in mediating mechanisms. Therefore, active sleep education interventions can improve the sleep quality of university students and have beneficial effects on reducing depression.

## Data Availability

The raw data supporting the conclusions of this article will be made available by the authors, without undue reservation.
